# The genera in the third catalogue (1836–1837) of Dejean’s Coleoptera collection

**DOI:** 10.3897/zookeys.282.4402

**Published:** 2013-04-02

**Authors:** Yves Bousquet, Patrice Bouchard

**Affiliations:** 1 Canadian National Collection of Insects, Arachnids and Nematodes, Agriculture and Agri-Food Canada, 960 Carling Avenue, Ottawa, Ontario, K1A 0C6, Canada

**Keywords:** Insecta, Coleoptera, nomenclature, Dejean, third catalogue, genus-group names, type species

## Abstract

All genus-group names first proposed or made available for the first time in the third edition of Dejean’s catalogue of his beetle collection are recorded. The following 18 names are made available for the first time in Dejean’s third catalogue: *Batoscelis* Dejean [Carabidae], *Laphyra* Dejean [Carabidae], *Sauriodes* Dejean [Staphylinidae], *Abrobapta* Dejean [Buprestidae], *Selagis* Dejean [Buprestidae], *Eurhipis* Dejean [Eucnemidae], *Anaeretes* Dejean [Scarabaeidae], *Doryscelis* Dejean [Scarabaeidae], *Epilissus* Dejean [Scarabaeidae], *Hoploscelis* Dejean [Scarabaeidae], *Brachygenius* Dejean [Tenebrionidae], *Capnisa* Dejean [Tenebrionidae], *Heterocheira* Dejean [Tenebrionidae], *Selenomma* Dejean [Tenebrionidae], *Dactylocrepis* Dejean [Curculionidae], *Lophodes* Dejean [Curculionidae], *Heterarthron* Dejean [Bostrichidae] and *Homalopus* Chevrolat [Chrysomelidae]. *Eurhipis* Dejean, 1836 is considered a junior synonym of *Phyllocerus* Lepeletier and Audinet-Serville, 1825 for the first time. *Abrobapta* Dejean, 1836 has precedence over *Torresita* Harold, 1869.

## Introduction

The catalogues of Dejean’s beetle collection (1821, 1833–1836a, 1836b–1837) are important works from a nomenclatural point of view but have been the source of much confusion in the literature. Coleopterists in the past have treated the new genus-group names included in those catalogues as *nomina nuda* since none of them are formally described. However, Dejean included one or more available species-group names in several of these genera, which made them available by indication (ICZN 1999: Article 12.2.5).

Silfverberg (1983, 1984a, 1984b) summarized nomenclatural information about the genus-group names introduced by Dejean in the first edition of his catalogue published in 1821 and Bousquet and Bouchard (2013) reviewed the names included in the second edition of Dejean’s catalogue published between 1833 and 1836. The objective of this paper is to summarize, for the first time, the nomenclatural status of all genus-group names listed in the third catalogue of Dejean’s Coleoptera collection published in 1836 and 1837.

## Dates of publication

The third catalogue of Dejean’s beetle collection was published in five livraisons. As there are no dates included in the published work itself, we have determined the earliest dates of publication for the livraisons as follows. For nomenclatural purposes, livraisons 1 to 4 [pp. 1–384] were published on the 30^th^ of July 1836 and livraison 5 [pp. i-xiv + 385–503] was published on the 27^th^ of May 1837 as determined by entries in the *Bibliographie de la France*, a journal published once a week detailing dates of receipts for works deposited in the National Library of France. As mentioned in Bousquet and Bouchard (2013), the sections containing the *chrysomélines* and *trimères* in the third catalogue of Dejean’s collection contain exactly the same information as in the same sections published in the second catalogue of Dejean’s collection published in 1836 (except pages numbers, which are different in the two publications).

## Precedence

Three publications issued published in 1836 and 1837 have important nomenclatural significance for the names that appear in Dejean’s third catalogue. These works either contain genus-group names that take precedence over names included in Dejean’s catalogue or include new species-group names listed in Dejean’s catalogue. In order to establish precedence, we have ascertained the dates of publication of these works.

[1] Schönherr, C.J. 1836. *Genera et species curculionidum, cum synonymia hujus familiae; species novae aut hactenus minus cognitae, descriptionibus A Dom. Leonardo Gyllenhal, C. H. Boheman, et entomologis aliis illustratae. Tomus tertius. Pars secunda*. Roret, Parisiis. Pp. 506–858. This book was presented to the *Société Entomologique de France* on 16 March 1836 (Ann. Soc. Ent. Fr. 5: xx) which precedes the publication of the first four livraisons of Dejean’s third catalogue recorded on 30 July 1836.

[2] Solier, A.J.J. 1836. Essai sur les collaptérides (suite). *Annales de la Société Entomologique de France* 5: 5–200, pls i-iv. Solier’s paper appeared in the first issue of the fifth volume of the *Annales*. This issue was recorded on 16 May 1836 by the *Académie des Sciences* (France) and so is considered to precede the first four livraisons of Dejean’s third catalogue recorded on 30 July 1836.

[3] Solier, A.J.J. 1837. Description d’une nouvelle espèce du genre *Cryptocephalus*. *Annales de la Société Entomologique de France* 5 [1836]: 687–688. This paper was published in the fourth and last issue of the fifth volume of the *Annales* which was recorded on 6 March 1837 by the *Académie des Sciences* (France). Solier’s publication was therefore published before livraison 5 of Dejean’s catalogue recorded on 27 May 1837.

## Methods

We summarize the nomenclatural status of all genus-group names first proposed or made available for the first time in the third edition of Dejean’s catalogue of his beetle collection. Names cited in Dejean’s second and third catalogues that were made available between the publication of Dejean’s second and third catalogues are listed in Table 1. Available names listed in Dejean’s third catalogue but not included in his previous catalogues are listed in Table 2. For each genus-group name first made available in Dejean’s third catalogue, we have determined the originally included available species, their type species and their current status. The methods used here are the same as those used in Bousquet and Bouchard (2013) and they are only briefly outlined here.

Originally included available species are those cited by name that were available at the time of publication of the livraisons of Dejean’s catalogue, whether they were listed as valid or invalid. A species’ name followed by a question mark in Dejean’s catalogue indicates that Dejean was uncertain if he had the right taxon. Such species are considered as “*species inquirenda*” and deemed not to be originally included (ICZN 1999: Article 67.2.5); they cannot be selected as type species. We have considered all species-group names attributed by Dejean to himself as unavailable except in the following two cases. First, when an author, prior to the publication of Dejean’s catalogue, proposed an identical specific name that he attributed to Dejean; second, when an author, prior to the publication of Dejean’s catalogue, proposed an identical specific name and stipulated that the specimen(s) was in the collection of Dejean. These are available species-group names in Dejean’s catalogue but are attributed to the authors that made them available earlier. We have assumed correct identity for all available species-group names listed in Dejean’s catalogue. The same major divisions used in Dejean’s catalogue (Pentamères, Hétéromères, Tétramères, Trimères and Dimères and their subdivisions) are used in this publication.

**Table 1.** Names cited in Dejean’s second and third catalogues that were made available between the publication of Dejean’s second and third catalogues. A number in square brackets following the year of publication refers to the publication treated in the “Precedence” section where priority between the publication and Dejean’s catalogue is established.

**Names in Dejean’s catalogue [year and page in Dejean]**	**Current names with author and date**
**Pentamères: Sternoxes**	
*Isorhipis* Lacordaire [1836*b*: 95]	*Isorhipis* Lacordaire, 1835
*Polybothris* Dejean [1836*b*: 78]	*Polybothris* Dupont, 1833 **^1^**
**Pentamères: Lamellicornes**	
*Aplonycha* Dejean [1836*b*: 179]	*Aplonycha* Boisduval, 1835 **^2^**
*Caelidia* Dejean [1836*b*: 172]	*Caelidia* Boisduval, 1835
*Geobatus* Dejean [1836*b*: 164]	*Geobatus* Boisduval, 1835
*Macrothops* Mac Leay [1836*b*: 181]	*Macrothops* Boisduval, 1835
*Sericesthis* Dejean [1836*b*: 181]	*Sericesthis* Boisduval, 1835
*Xylonichus* Mac Leay [1836*b*: 172]	*Xylonichus* Boisduval, 1835
**Hétéromères: Mélasomes**	
*Cilibe* Latreille [1836*b*: 208]	*Celibe* Boisduval, 1835
*Pachycoelia* Boisduval [1836*b*: 208]	*Pachycoelia* Boisduval, 1835
*Pachyscelis* Solier [1836*b*: 198]	*Pachyscelis* Solier, 1836 [**2**]
*Pterolasia* Solier [1836*b*: 198]	*Pterolasia* Solier, 1836 [**2**]
**Hétéromères: Ténébrionites**	
*Baryscelis* Boisduval [1836*b*: 226]	*Baryscelis* Boisduval, 1835
**Hétéromères: Hélopiens**	
*Atractus* Mac Leay [1836*b*: 233]	*Atractus* Boisduval, 1835
**Tétramères: Curculionites**	
*Anchylorhynchus* Klug [1836*b*: 305]	*Anchylorhynchus* Schönherr, 1836 [**1**]
*Aporhina* Boisduval [1836*b*: 262]	*Aporhina* Boisduval, 1835

^1^ Dupont originally proposed the name under the spelling *Polybotris*. An application to the Commission by Bouchard et al. (in press) was recently submitted to conserve the spelling *Polybothris* which is in prevailing usage.^2^ This name was proposed in synonymy with *Melolontha* by Boisduval (1835: 193). It is available because it was treated before 1961 as an available name and adopted as the name of a taxon (e.g., Duponchel and Chevrolat 1841: 16) (ICZN 1999: Article 11.6.1). This name is considered an invalid synonym of *Colpochila* Erichson, 1843 (Smith 2006: 166, as “*Haplonycha* Dejean, 1836”) but has priority. Reversal of Precedence or an application to the Commission is necessary to retain *Colpochila* Erichson as the valid name.

**Table 2.** Available names listed in Dejean’s third catalogue but not included in his previous catalogues. A number in square brackets following the year of publication refers to the publication treated in the “Precedence” section where priority between the publication and Dejean’s catalogue is established.

**Names in Dejean’s catalogue [year and page in Dejean]**	**Current names with author and date**
**Pentamères: Carabiques**	
*Arsinoe* De Laporte [1836*b*: 13]	*Arsinoe* Laporte, 1834
*Chalybe* De Laporte [1836*b*: 56]	*Calybe* Laporte, 1834
*Cnemacanthus* Gray [1836*b*: 43]	*Cnemacanthus* Gray, 1832
*Microcheila* Brullé [1836*b*: 46]	*Microcheila* Brullé, 1834
*Omalosoma* Mac Leay [1836*b*: 37]	*Omalosoma* Gory, 1833
*Scolytus* Fabricius [1836*b*: 26]	*Scolytus* Fabricius, 1790
**Pentamères: Hydrocanthares**	
*Copelatus* Erichson [1836*b*: 63]	*Copelatus* Erichson, 1832
*Cybister* Curtis [1836*b*: 60]	*Cybister* Curtis, 1827
*Gyretes* Brullé [1836*b*: 67]	*Gyretes* Brullé, 1835
*Paelobius* Schönherr [1836*b*: 64]	*Paelobius* Schönherr, 1808
**Pentamères: Brachélytres**	
*Bryocharis* Lacordaire [1836*b*: 79]	*Bryocharis* Lacordaire, 1835
*Gyrohypnus* Kirby [1836*b*: 72]	*Gyrohypnus* Leach, 1819
*Leptochirus* Germar [1836*b*: 76]	*Leptochirus* Germar, 1824
*Lesteva* Latreille [1836*b*: 77]	*Lesteva* Latreille, 1797
*Physetops* Mannerheim [1836*b*: 71]	*Physetops* Mannerheim, 1830
*Siagonium* Kirby [1836*b*: 76]	*Siagonium* Kirby & Spence, 1815
**Pentamères: Malacodermes**	
*Brachymorphus* Chevrolat [1836*b*: 128]	*Brachymorphus* Chevrolat, 1835
*Dascillus* Latreille [1836*b*: 109]	*Dascillus* Latreille, 1797
*Eucynetus* Schüppel [1836*b*: 110]	*Eucinetus* Germar, 1818
*Telephorus* Schaeffer [1836*b*: 118]	*Telephorus* Schaeffer, 1766
**Pentamères: Terediles**	
*Necrobia* Latreille [1836*b*: 127]	*Necrobia* Olivier, 1795
*Opilo* Latreille [1836*b*: 126]	*Opilo* Latreille, 1802
*Thanasimus* Latreille [1836*b*: 127]	*Thanasimus* Latreille, 1806
**Pentamères: Clavicornes**	
*Catheretes* Herbst [1836*b*: 136]	*Kateretes* Herbst, 1793
*Choleva* Latreille [1836*b*: 133]	*Choleva* Latreille, 1797
*Dacne* Latreille [1836*b*: 137]	*Dacne* Latreille, 1797
*Dryops* Olivier [1836*b*: 146]	*Dryops* Olivier, 1791
*Limnius* Illiger [1836*b*: 145]	*Limnius* Illiger, 1802
*Monomma* Klug [1836*b*: 144]	*Monomma* Klug, 1833
*Sarapus* Fischer [1836*b*: 133]	*Sarapus* Fischer von Waldheim, 1821
**Pentamères: Lamellicornes**	
*Agestrata* Eschscholtz [1836*b*: 189]	*Agestrata* Eschscholtz, 1829
*Chiasognathus* Stéphens [1836*b*: 193]	*Chiasognathus* Stephens, 1832
*Deltochilum* Eschscholtz [1836*b*: 151]	*Deltochilum* Eschscholtz, 1822
**Hétéromères: Mélasomes**	
*Amophorus* Guérin [1836*b*: 203]	*Ammophorus* Guérin-Méneville, 1831
*Anatolica* Eschscholtz [1836*b*: 205]	*Anatolica* Eschscholtz, 1831
*Dailognatha* Stéven [1836*b*: 205]	*Dailognatha* Eschscholtz, 1831
*Evaniosomus* Guérin [1836*b*: 204]	*Evaniosomus* Guérin-Méneville, 1834
*Gyriosoma* Guérin [1836*b*: 206]	*Gyriosomus* Guérin-Méneville, 1834
*Hylithus* Guérin [1836*b*: 204]	*Hylithus* Guérin-Méneville, 1834
*Hyperops* Eschscholtz [1836*b*: 203]	*Hyperops* Eschscholtz, 1831
*Mesostena* Eschscholtz [1836*b*: 205]	*Mesostena* Eschscholtz, 1831
*Pachychila* Eschscholtz [1836*b*: 206]	*Pachychila* Eschscholtz, 1831
**Hétéromères: Ténébrionites**	
*Dolichoderus* Klug [1836*b*: 227]	*Dolichoderus* Klug, 1833
*Nycteropus* Klug [1836*b*: 227]	*Nycteropus* Klug, 1833
**Tétramères: Curculionites**	
*Antliarhinus* Schönherr [1836*b*: 267]	*Antliarhinus* Schönherr, 1826
*Celetes* Schönherr [1836*b*: 311]	*Celetes* Schönherr, 1836 [**1**]
*Choragus* Kirby [1836*b*: 259]	*Choragus* Kirby, 1819
*Coleomerus* Schönherr [1836*b*: 316]	*Coleomerus* Schönherr, 1836 [**1**]
*Deracanthus* Schönherr [1836*b*: 269]	*Deracanthus* Schönherr, 1823
*Eupages* Schönherr [1836*b*: 287]	*Eupages* Schönherr, 1833
*Strongylotes* Schönherr [1836*b*: 310]	*Strongylotes* Schönherr, 1836 [**1**]
**Tétramères: Longicornes**	
*Ceraegidion* Boisduval [1836*b*: 372]	*Ceraegidion* Boisduval, 1835
*Dorysthetus* Vigors [1836*b*: 341]	*Dorysthenes* Vigors, 1826

## List of genus-group names in Dejean’s third catalogue (1836–1837)

Below is a list of genus-group names first proposed or first made available in Dejean’s third catalogue. Many generic names were proposed for the first time in Dejean’s third catalogue as invalid synonyms. According to the ICZN (1999: Article 11.6.1), a name originally published as junior synonym of an available name can be available from its first publication as synonym if it had been treated before 1961 as an available name and either adopted as the name of a taxon or treated as a senior homonym. The originally included species are the species (cited by available names) first directly associated with the synonym (ICZN 1999: Article 67.12). In several instances Dejean proposed a new generic name as valid while he listed in synonymy a genus name which was already available. In those cases we have concluded that Dejean proposed replacement names.

### Pentamères: Carabiques

***Actena* Dejean, 1836b: 12**

Originally included available species: none.

***Adrimus* Dejean, 1836b: 37**

Originally included available species: none.

***Batoscelis* Dejean, 1836b: 46**

Originally included available species: *Agonoderus discipennis* Dejean, 1831; *Agonoderus oblongus* Dejean, 1831.

Type species: *Agonoderus oblongus* Dejean, 1831 by subsequent designation (Noonan 1976: 25).

Current status: valid genus in Carabidae (*fide* Lorenz 2005: 361).

***Batrachion* Dejean, 1836b: 50** (as “Batrachion. *Chevrolat*.”)

Originally included available species: none.

***Ericatus* Dejean, 1836b: 47** (as “Ericatus. *Dupont*.”)

Originally included available species: none.

***Euchlamys* Dejean, 1836b: 43**

Originally included available species: none.

***Laphyra* Dejean, 1836b: 6** (as “Laphyra. *Dupont*.”)

Originally included available species: *Cicindela audouinii* Barthélemy, 1835.

Type species: *Cicindela audouinii* Barthélemy, 1835 (= *Cicindela ritchiei* Vigors, 1825) by monotypy.

Current status: junior homonym of *Laphyra* Billberg, 1820 [Diptera]; senior objective synonym of *Neolaphyra* Bedel, 1895 in Carabidae (*fide* Puchkov and Matalin 2003: 114).

***Listropus* Dejean, 1836b: 17** (as “Listropus. *Audouin*.”)

Originally included available species: none.

***Ocydromus* Dejean, 1836b: 27**

Originally included available species: none.

Comments. This name is treated as different than *Ocydromus* Clairville, 1806 in Carabidae.

***Oxycrepis* Dejean, 1836b: 37**

Originally included available species: none.

***Psilocera* Dejean, 1836b: 6** (as “Psilocera. *Brullé*.”)

Originally included available species: none.

***Tachybaenus* Dejean, 1836b: 6** (as “Tachybaenus. Dupont.”)

Comments. This name was listed by Dejean as an invalid synonym of *Psilocera* Dejean, 1836, a *nomen nudum*. Therefore, the name is not available.

### Pentamères: Brachélytres

***Bolitogyrus* Dejean, 1836b: 76**

Originally included available species: none.

***Leiosoma* Dejean, 1836b: 76** (as “Leiosoma. *Chevrolat*.”)

Originally included available species: none.

***Mycetrupes* Dejean, 1836b: 67**

Originally included available species: none.

***Sauriodes* Dejean, 1836b: 72**

Originally included available species: *Staphylinus alternans* Gravenhorst, 1802; *Staphylinus fulminans* Gravenhorst, 1802; *Staphylinus melanocephalus* Gravenhorst, 1806; *Staphylinus pilicornis* Paykull, 1790 (as “Var. *Pilicornis*. *Gyllenhal*.”).

Type species: *Staphylinus fulminans* Gravenhorst, 1802 (= *Staphylinus punctulatus* Goeze, 1777) by subsequent designation (Blackwelder 1952: 344).

Current status: junior synonym of *Othius* Stephens, 1829 in Staphylinidae (*fide* Chevrolat 1847: 240).

### Pentamères: Sternoxes

***Abrobapta* Dejean, 1836b: 90**

Originally included available species: *Buprestis chrysoptera* Boisduval, 1835 (as “Chrysoptera. *Latreille*.”); *Buprestis viridinitens* Boisduval, 1835 (as “Viridinitens. *Dej*.”).

Type species: *Buprestis chrysoptera* Boisduval, 1835 (= *Buprestis cuprifera* Kirby, 1819) by subsequent designation (Duponchel 1839: 18).

Current status: senior synonym of *Torresita* Harold, 1869 in Buprestidae (*fide* Bellamy 2002: 110).

Comments. *Abrobapta* Dejean, 1836 has precedence over *Torresita* Harold, 1869 which is currently used as valid (e.g., Bellamy 2008: 1303). Reversal of Precedence (ICZN 1999: Article 23.9) or an application to the Commission is necessary to conserve usage of the name *Torresita* Harold.

***Arhipis* Dejean, 1836b: 95**

Originally included available species: none.

***Crepicardus* Dejean, 1836b: 103**

Originally included available species: none.

***Eurhipis*** **Dejean, 1836b: 96**

Originally included available species: *Eucnemis ramicornis* Latreille, 1834 (as “Ramicornis. *Klug*.”).

Type species: *Eucnemis ramicornis* Latreille, 1834 by monotypy.

Current status: junior homonym of *Eurhipis* Laporte, 1834 [Rhipiceridae]; junior subjective synonym of *Phyllocerus* Lepeletier and Audinet-Serville, 1825 in Eucnemidae (**new synonymy**).

***Euryotes* Dejean, 1836b: 92**

Originally included available species: none.

***Selagis* Dejean, 1836b: 89**

Originally included available species: *Buprestis caloptera* Boisduval, 1835 (as “Caloptera. *Mac Leay*.”).

Type species: *Buprestis caloptera* Boisduval, 1835 by monotypy.

Current status: senior objective synonym of *Curis* Gory and Laporte, 1837 in Buprestidae (*fide* Bellamy 2008: 1292).

Comments. This name has precedence over *Curis* Gory and Laporte. The ICZN (2008) rejected an application to conserve *Curis* as a valid name by suppressing “*Selagis* Mannerheim, 1837.” The correct name for the genus should be *Selagis* Dejean, 1836.

### Pentamères: Malacodermes

***Eurypalpus* Dejean, 1836b: 109**

Originally included available species: none.

***Meconyx* Dejean, 1836b: 125** (as “Meconyx. *Schönherr*.”)

Originally included available species: none.

### Pentamères: Clavicornes

***Eurytarsus* Dejean, 1836b: 133**

Originally included available species: none.

### Pentamères: Lamellicornes

***Anaeretes* Dejean, 1836b: 181**

Originally included available species: *Melolontha elongata* Fabricius, 1792 (as “elongata. *Say. Fabr.?*”); *Melolontha elongatula* Schönherr, 1817.

Type species: *Melolontha elongata* Fabricius, 1792 (=*Melolontha elongatula* Schönherr, 1817) by monotypy.

Current status: junior synonym of *Dichelonyx* Harris, 1827 in Scarabaeidae (*fide* Evans 2003: 258).

Comments: The name *elongatula* is listed in synonymy with *elongata* in Dejean’s catalogue; therefore the type species of *Anaeretes* is *elongata* by monotypy (ICZN 1999: Article 68.3).

***Atimus* Dejean, 1836b: 165**

Originally included available species: none.

***Coryptius* Dejean, 1836b: 194**

Originally included available species: none.

***Doryscelis* Dejean, 1836b: 189**

Originally included available species: *Cetonia calcarata* Klug, 1833; *Cetonia inscripta* Gory & Percheron, 1835 (as “*Inscripta. Latreille*.”).

Type species: *Cetonia calcarata* Klug, 1833 by monotypy.

Current status: valid genus in Scarabaeidae (*fide* Antoine 1989: 199, as “*Doryscelis* Burmeister, 1842”).

Comments: The name *inscripta* is listed in synonymy with *calcarata* in Dejean’s catalogue; therefore the type species of *Doryscelis* is *calcarata* by monotypy (ICZN 1999: Article 68.3).

***Epilissus* Dejean, 1836b: 151**

Originally included available species: *Canthon prasinus* Klug, 1833; *Coprobius viridis* Guérin-Méneville, 1830 (as “*Viridis. Latreille*.”).

Type species: *Canthon prasinus* Klug, 1833 by subsequent designation (Reiche 1841: 212).

Current status: valid genus in Scarabaeidae (*fide* Montreuil 2011: 104, as “*Epilissus* Reiche, 1841”).

***Hoploscelis* Dejean, 1836b: 184**

Originally included available species: *Scarabaeus crassipes* Olivier, 1789; *Trichius grossipes* Schönherr, 1817.

Type species: *Trichius grossipes* Schönherr, 1817 by monotypy.

Current status: junior homonym of *Hoploscelis* Audinet-Serville, 1832 [Cerambycidae]; senior subjective synonym of *Hoplocnemis* Harold, 1869 in Scarabaeidae (*fide* Schein 1959: 149, as “Hoploscelis Burm[eister]”).

Comments. The name *crassipes* is listed in synonymy with *grossipes* in Dejean’s catalogue; therefore the type species of *Hoploscelis* is *grossipes* by monotypy (ICZN 1999: Article 68.3).

***Microdoris* Dejean, 1836b: 184**

Originally included available species: none.

***Rhizonemus* Dejean, 1836b: 180**

Originally included available species: none.

***Scelophysa* Dejean, 1836b: 183**

Originally included available species: none.

## Hétéromères: Mélasomes

***Ancylognathus* Dejean, 1836b: 201**

Originally included available species: none.

***Brachygenius* Dejean, 1836b: 206** (as “Brachygenius. *Solier*.”)

Comments. This genus is treated as an unnecessary replacement name for *Gyriosomus* Guérin-Méneville, 1834 (as “*Gyriosoma* Guérin”) in Tenebrionidae.

***Capnisa* Dejean, 1836b: 197**

Originally included available species: *Bradyus karelini* Faldermann, 1836.

Type species: *Bradyus karelini* Faldermann, 1836 by monotypy.

Current status: junior subjective synonym of *Gnathosia* Fischer von Waldheim, 1821 in Tenebrionidae (*fide* Löbl et al. 2008: 190).

Comments. The date of publication of volume 9 of the *Bulletin de la Société Impériale des Naturalistes de Moscou*, where the species *Bradyus karelini* Faldermann was published, is unknown besides the year. The permit for publication was delivered on 17 January 1836 [Julian calendar = 29 January 1836 for the Gregorian calendar] and suggests that the actual date of publication preceeds that of the first four livraisons of Dejean’s catalogue recorded on 30 July 1836. *Capnisa* was considered as available and credited to Dejean in the recent *Catalogue of Palaearctic Coleoptera* (Löbl et al. 2008: 190). To promote stability we consider that *Bradyus karelini* Faldermann, 1836 was available before the publication of Dejean’s catalogue despite that the date of publication of the *Bulletin* should theoretically be the last day of the year (ICZN 1999: 21.3.2). An application to the Commission may be necessary in this case.

***Selenomma* Dejean, 1836b: 203** (as “Selenomma. *Solier*.”)

Comments. This genus is treated as an unnecessary replacement name for *Ammophorus* Guérin-Méneville, 1831 (as “*Amophorus* Guérin”) in Tenebrionidae.

***Zopherus* Dejean, 1836b: 207** (as “Zopherus. *Gray*.”)

Originally included available species: none.

### Hétéromères: Taxicornes

***Heterocheira* Dejean, 1836b: 220**

Originally included available species: *Uloma australis* Boisduval, 1835 (as “Australis. *Dej*.”).

Type species: *Uloma australis* Boisduval, 1835 by monotypy.

Current status: valid genus in Tenebrionidae (*fide* Matthews and Bouchard 2008: 322).

***Peneta* Dejean, 1836b: 221**

Originally included available species: none.

***Prosomenes* Dejean, 1836b: 216**

Originally included available species: none.

### Tétramères: Curculionites

***Cateschnus* Dejean, 1836b: 327** (as “Cateschnus. *Schönherr*.”)

Originally included available species: none.

***Catolethrus* Dejean, 1836b: 330** (as “Catolethrus. *Schönherr*.”)

Originally included available species: none.

***Catomismus* Dejean, 1836b: 302** (as “Catomismus. *Schönherr*.”)

Originally included available species: none.

***Cladobius* Dejean, 1836b: 303**

Originally included available species: none.

***Dactylocrepis* Dejean, 1836b: 315**

Originally included available species: *Baris bidens* Boheman, 1836 (as “Var. *Bidens*. *Dej*.”); *Cylindrocerus flabellitarsis* Boheman, 1836 (as “Flabellitarsis. *Chevrolat. Schönherr*. (Cylindrocerus.)”).

Type species: *Cylindrocerus flabellitarsis* Boheman, 1836 by monotypy.

Current status: valid genus in Curculionidae (*fide* Alonso-Zarazaga and Lyal 1999: 98).

Comments. The variety *bidens* is listed in synonymy with *flabellitarsis* in Dejean’s catalogue; therefore the type species of *Dactylocrepis* is *flabellitarsis* by monotypy (ICZN 1999: Article 68.3). The two species listed under this genus name were described in 1836 by Boheman [in Schönherr] and were made available before the publication of Dejean’s third catalogue (see “Precedence” section[1]).

***Haplurus* Dejean, 1836b: 306** (as “Haplurus. *Schönherr*.”)

Originally included available species: none.

***Lophodes* Dejean, 1836b: 284** (as “Lophodes. *Schönherr*.”)

Originally included available species: *Lophotus nodipennis* Hope, 1834 (as “Nodipennis. *Schönherr*.”)

Type species: *Lophotus nodipennis* Hope, 1834 by monotypy.

Current status: junior subjective synonym of *Psuchocephalus* Latreille, 1828 in Curculionidae (*fide* Alonso-Zarazaga and Lyal 1999: 140). The Commission (ICZN 2004) has ruled that the name *Aegorhinus* Erichson, 1834 is given priority over the name *Psuchocephalus* Latreille, 1828 whenever the two are considered to be synonyms.

***Menemachus* Dejean, 1836b: 311** (as “Menemachus. *Schönherr*.”)

Originally included available species: none.

***Rachys* Dejean, 1836b: 271** (as “Rachys. *Hope*.”)

Originally included available species: none.

***Trichocorynus* Dejean, 1836b: 319**

Originally included available species: none.

### Tétramères: Xylophages

***Amphibolonarzon* Dejean, 1836b: 339** (as “Amphibolonarzon. *Porro*.”)

Comments. This name was first proposed as an invalid synonym of *Calyptobium* Dejean, 1836, a *nomen nudum*. Therefore, the name is not available.

***Calyptobium* Dejean, 1836b: 339** (as “Calyptobium. *Villa*.”)

Originally included available species: none.

***Heterarthron* Dejean, 1836b: 334** (as “Heterarthron. *Guérin*.”).

Originally included available species: *Apate femoralis* Fabricius, 1792 (as “*Femoralis. Olivier*.”); *Apate gonagra* Fabricius, 1798.

Type species: *Apate gonagra* Fabricius, 1798 by monotypy.

Current status: junior objective synonym of *Melalgus* Dejean, 1835 in Bostrichidae (*fide* Ivie 2002: 239).

Comment. This name was first proposed by Dejean as an invalid synonym of *Melalgus* Dejean, 1835. It is available because it was treated before 1961 as an available name and adopted as the name of a taxon (e.g., Guérin-Méneville 1844: 186).

The name *femoralis* is listed in synonymy with *gonagra* in Dejean’s catalogue; therefore the type species of *Heterarthron* is *gonagra* by monotypy (ICZN 1999: Article 68.3).

### Tétramères: Longicornes

***Aegorhinus* Dejean, 1836b: 360**

Originally included available species: none.

***Cereopsis* Dejean, 1836b: 370** (as “Cereopsis. *Dupont*.”)

Originally included available species: none.

***Charientoptenus* Dejean, 1836b: 346** (as “Charientoptenus. *Chevrolat*.”)

Comments. This name was listed by Dejean as an invalid synonym of *Sphenothecus* Dejean, 1835, a *nomen nudum*. Therefore, the name is not available.

***Enaphalodes* Dejean, 1836b: 352** (as “Enaphalodes. *Chevrolat*.”)

Originally included available species: none.

***Hemicladus* Dejean, 1836b: 373**

Originally included available species: none.

***Onchoderes* Dejean, 1836b: 377** (as “Onchoderes. *Chevrolat*.”)

Originally included available species: none.

***Trichoderes* Dejean, 1836b: 343** (as “Trichoderes. *Chevrolat*.”)

Originally included available species: none.

***Trigonarthris* Dejean, 1836b: 383**

Comments. This name was proposed as a replacement name for *Trigonotarsis* Dejean, 1835, a *nomen nudum*. Therefore, *Trigonarthris* is also a *nomen nudum*.

***Tropipleurites* Dejean, 1836b: 371** (as “Tropipleurites. *Chevrolat*.”)

Comments. This name was listed by Dejean as an invalid synonym of *Dorcacephalum* Dejean, 1835, a *nomen nudum*. Therefore, the name is not available.

### Tétramères: Chrysomélines

***Homalopus* Chevrolat, 1837: 446**

Originally included available species: *Cryptocephalus loreyi* Solier, 1837 (as “Loreyi. *Dej*.”).

Type species: *Cryptocephalus loreyi* Solier, 1837 by monotypy.

Current status: senior subjective synonym of *Heterichnus* Warchałowski, 1991 in Chrysomelidae (*fide* Lopatin et al. 2010: 604).

Comments. *Homalopus* Chevrolat, 1837 has precedence over *Heterichnus* Warchałowski, 1991 and should be used as valid as was done by several authors (e.g., Warchałowski 1991; Steinhausen 2007). *Homalopus* Chevrolat, 1837 was treated as a junior homonym of the tenebrionid name *Homalopus* Solier (1836: 78) by Lopatin et al. (2010: 604) but Solier’s generic name is unavailable. Solier’s species-group taxon, *Cryptocephalus loreyi*, was made available before the publication of Dejean’s third catalogue (see Precedence section[3]).
